# Eight years of throughfall exclusion reshaped bacterial, fungal, and arbuscular mycorrhizal fungal communities in managed forest soil

**DOI:** 10.1128/mra.00209-26

**Published:** 2026-06-09

**Authors:** Nobuhiko Shigyo, Keizo Hirai

**Affiliations:** 1 Department of Forest Soils, Forestry and Forest Products Research Institute 57880, Tsukuba, Ibaraki, Japan; California State University San Marcos, San Marcos, California, USA

**Keywords:** amplicon sequencing, arbuscular mycorrhizal fungi, drought, forest soil, soil bacteria, soil fungi, throughfall exclusion

## Abstract

Forest soils support tree productivity and resilience. However, long-term manipulations of drought and substrate inputs are rare. This resource reports 16S rRNA gene, ITS2, and long-amplicon (SSU–ITS–LSU) sequencing datasets of bacterial, fungal, and arbuscular mycorrhizal fungal communities from Japanese forest soil following 8 years of throughfall exclusion.

## ANNOUNCEMENT

Drought alters soil microbial communities and associated carbon and nutrient dynamics (1). Although short-term throughfall exclusion experiments are common (2), long-term manipulations are limited. We sequenced bacterial, fungal, and arbuscular mycorrhizal fungal (AMF) communities in soil from an 8-year throughfall exclusion experiment in a managed forest. These data sets support studies of drought-induced shifts in forest soil microbiomes.

Soil was sampled in October 2025 at the Chiyoda Experimental Station of the Forestry and Forest Products Research Institute, Japan (36°10′57.4″ N, 140°13′01.2″ E). This site has been roofed continuously since 2017 (2 m aboveground) to exclude rainfall and litterfall (3). Surface mineral soils (0–10 cm) were collected from four points per treatment (rainfall/litter exclusion and adjacent control), transported on ice, and stored at −80°C.

For bacterial and fungal community profiling, frozen soil samples were freeze-dried, bead-homogenized, and used for DNA extraction with a Lab-Aid 824s Kit (ZEESAN, Xiamen, China). DNA was quantified fluorometrically. The V4 region of the 16S rRNA gene was amplified with primers 515F/806R (5′-GTGCCAGCMGCCGCGGTAA-3′/5′-GGACTACHVGGGTWTCTAAT-3′) (4) and the fungal ITS2 region with gITS7/ITS4 (5′-GTGARTCATCGARTCTTTG-3′/5′-TCCTCCGCTTATTGATATGC-3′) (5). Amplicon libraries were prepared by two-step tailed PCR using KOD FX Neo DNA polymerase (Toyobo, Osaka, Japan); first-round PCR comprised 94°C for 2 min; 30 cycles of 98°C for 10 s, 50°C for 30 s, and 68°C for 30 s for 16S, or 94°C for 15 s, 50°C for 30 s, and 68°C for 1 min for ITS2; and 68°C for 7 min. Libraries were sequenced on the Illumina NextSeq 1000 platform using 2 × 300 bp paired-end reads (Seibutsu Giken Inc., Kanagawa, Japan) (Table 1). Sequence processing followed previously described methods (6), using DADA2 v1.38.0 (7) in R v4.5.2 with truncLen = c(240, 220) for bacteria. Bacterial and fungal taxonomy was assigned against SILVA v138.1 and UNITE v10.0, respectively.

**TABLE 1:** Summary of sequencing data

Sample ID	No. of raw reads	BioSample accession no.	SRA accession no.
Ctrl1-V4	49,252	SAMD01817745	DRR907017
Ctrl2-V4	49,963	SAMD01817746	DRR907018
Ctrl3-V4	49,136	SAMD01817747	DRR907019
Ctrl4-V4	48,433	SAMD01817748	DRR907020
Roof1-V4	49,567	SAMD01817749	DRR907021
Roof2-V4	48,547	SAMD01817750	DRR907022
Roof3-V4	49,122	SAMD01817751	DRR907023
Roof4-V4	49,150	SAMD01817752	DRR907024
Ctrl1-gITS	49,231	SAMD01817753	DRR907025
Ctrl2-gITS	49,585	SAMD01817754	DRR907026
Ctrl3-gITS	49,716	SAMD01817755	DRR907027
Ctrl4-gITS	49,437	SAMD01817756	DRR907028
Roof1-gITS	49,575	SAMD01817757	DRR907029
Roof2-gITS	49,674	SAMD01817758	DRR907030
Roof3-gITS	49,025	SAMD01817759	DRR907031
Roof4-gITS	49,076	SAMD01817760	DRR907032
Ctrl1-AM	109,937	SAMD01817761	DRR907033
Ctrl2-AM	110,049	SAMD01817762	DRR907034
Ctrl3-AM	110,075	SAMD01817763	DRR907035
Ctrl4-AM	109,991	SAMD01817764	DRR907036
Roof1-AM	110,011	SAMD01817765	DRR907037
Roof2-AM	110,230	SAMD01817766	DRR907038
Roof3-AM	110,101	SAMD01817767	DRR907039
Roof4-AM	110,071	SAMD01817768	DRR907040

For AMF, genomic DNA was extracted from frozen soils using a NucleoSpin Soil Kit (Macherey-Nagel, Düren, Germany). Long-amplicon sequencing targeting the SSU–ITS–LSU region was performed using nested PCR (8) with primer sets NS31/LSUmAr13 + LSUmAr24 and AML1/LSUmAr13 + LSUmAr24 (9), followed by final amplification with NS31_Glo3/wLSUmBr (5′-TTGYTGCRGTTAAAAAGCTCG-3′/5′-AACACTCGCAYAYATGYTAGA-3′) (10). Detailed PCR conditions are available at protocols.io (11). Amplicons were purified using AMPure XP beads (Beckman Coulter Life Sciences, Brea, CA, USA) and pooled in equal volumes. Pooled amplicons were sequenced at Seibutsu Giken Inc. on a PacBio Revio platform (Pacific Biosciences, Menlo Park, CA, USA) (Table 1). Mean HiFi read length ranged from 2,502 to 2,644 bp across samples. PacBio CCS reads were trimmed with cutadapt v5.2, processed with ITSx v1.1.3 (12) and VSEARCH v2.30.4 (13), and clustered into 97% operational taxonomic units (OTUs). Taxonomy was assigned by BLASTn v2.16.0 against the Maarj*AM* (14) database (coverage ≥ 70%, identity ≥ 80%, *E* ≤ 1 × 10^−^²⁰). OTUs identified as Glomeromycota were retained; those with <90% identity were verified against the National Center for Biotechnology Information (NCBI) database.

**Fig 1:**
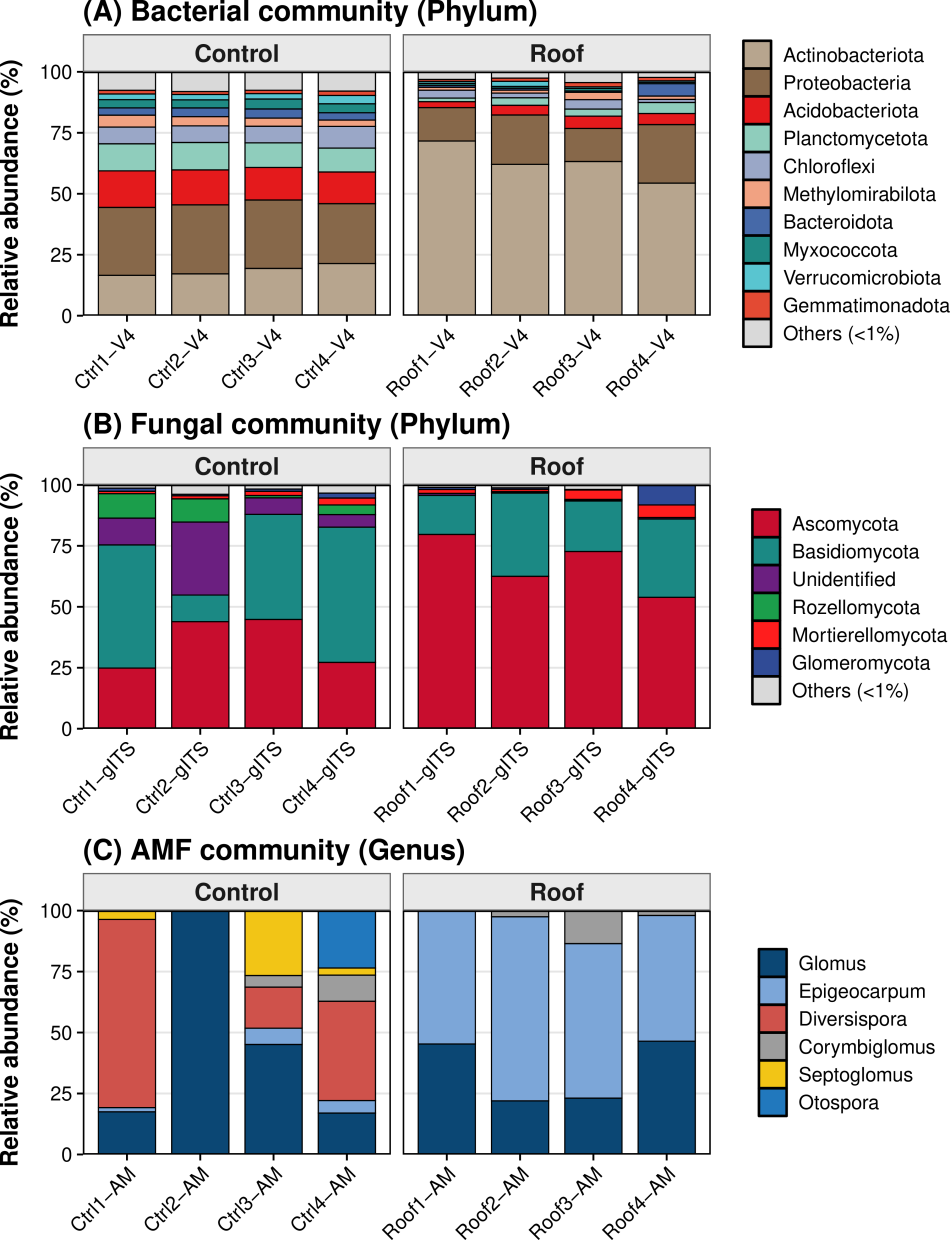
Relative abundance (%) of (**A**) bacterial phyla, (**B**) fungal phyla, and (**C**) arbuscular mycorrhizal fungal (AMF) genera in samples of control and throughfall exclusion forest soil. Each bar represents an individual soil sample (*n* = 4 per treatment). Taxa with a relative abundance of <1% across all samples were grouped into “Others (<1%).” Fungal taxa that could not be assigned to a known phylum were designated as “Unidentified.”

Dominant bacterial phyla were Actinobacteriota (control: 16.5%–21.4%, roof: 54.4%–71.6%), Proteobacteria (control: 24.6%–28.3%, roof: 13.6%–24.0%), and Acidobacteriota (control: 13.0%–14.9%, roof: 2.4%–5.0%; Fig. 1A). Major fungal phyla were Ascomycota (control: 24.9%–44.8%, roof: 53.9%–79.7%), Basidiomycota (control: 10.9%–55.5%, roof: 16.0%–34.1%), and unidentified phyla (control: 5.1%–29.9%, roof: 0.3%–0.8%; Fig. 1B). For AMF, prevalent genera were *Glomus* (control: 17.0%–99.9%, roof: 22.0%–46.5%), *Epigeocarpum* (control: 0.0%–6.7%, roof: 51.6%–75.5%), and *Diversispora* (control: 0.0%–77.2%, roof: 0.0%; Fig. 1C).

## Data Availability

The raw sequence data are available in the DNA Data Bank of Japan Sequence Read Archive under the BioProject accession number PRJDB40262.
